# Glycosphingolipids and neuroinflammation in Parkinson’s disease

**DOI:** 10.1186/s13024-020-00408-1

**Published:** 2020-10-17

**Authors:** Karim Belarbi, Elodie Cuvelier, Marie-Amandine Bonte, Mazarine Desplanque, Bernard Gressier, David Devos, Marie-Christine Chartier-Harlin

**Affiliations:** 1 Univ. Lille, Inserm, CHU-Lille, Lille Neuroscience & Cognition, 1 Place de Verdun, 59006 Lille, France; 2 Département de Pharmacologie de la Faculté de Pharmacie, Univ. Lille, Lille, France; 3 Département de Pharmacologie Médicale, I-SITE ULNE, LiCEND, Lille, France

**Keywords:** Gangliosides, Gaucher Disease, Glucocerebrosides, Glucosylceramides, Lipids, Microglia, Neurodegenerative Diseases, Parkinson Disease, Sphingolipids, Synucleinopathies

## Abstract

Parkinson's disease is a progressive neurodegenerative disease characterized by the loss of dopaminergic neurons of the nigrostriatal pathway and the formation of neuronal inclusions known as Lewy bodies. Chronic neuroinflammation, another hallmark of the disease, is thought to play an important role in the neurodegenerative process. Glycosphingolipids are a well-defined subclass of lipids that regulate crucial aspects of the brain function and recently emerged as potent regulators of the inflammatory process. Deregulation in glycosphingolipid metabolism has been reported in Parkinson’s disease. However, the interrelationship between glycosphingolipids and neuroinflammation in Parkinson’s disease is not well known. This review provides a thorough overview of the links between glycosphingolipid metabolism and immune-mediated mechanisms involved in neuroinflammation in Parkinson’s disease. After a brief presentation of the metabolism and function of glycosphingolipids in the brain, it summarizes the evidences supporting that glycosphingolipids (i.e. glucosylceramides or specific gangliosides) are deregulated in Parkinson’s disease. Then, the implications of these deregulations for neuroinflammation, based on data from human inherited lysosomal glycosphingolipid storage disorders and gene-engineered animal studies are outlined. Finally, the key molecular mechanisms by which glycosphingolipids could control neuroinflammation in Parkinson’s disease are highlighted. These include inflammasome activation and secretion of pro-inflammatory cytokines, altered calcium homeostasis, changes in the blood-brain barrier permeability, recruitment of peripheral immune cells or production of autoantibodies.

## Background

**Fig. 1:**
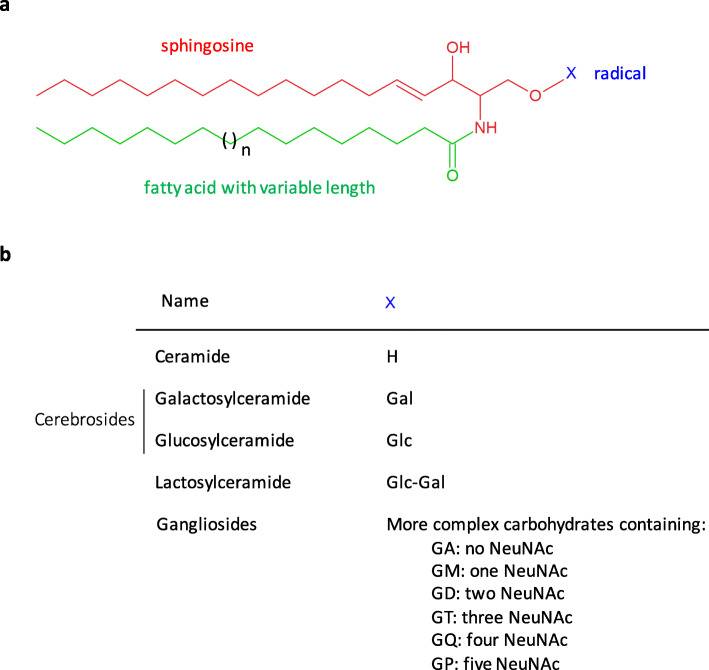
Basic chemical structure of glycosphingolipids. **a.** Glycosphingolipids are composed of a sphingosine, a fatty acid chain (these two forming a ceramide) and a carbohydrate moiety (X). The fatty acid attached to the sphingosine may vary in chain length and in degree of unsaturation and/or hydroxylation. **b.** Example of glycosphingolipids and their corresponding carbohydrate moiety. A sphingolipid with a X group consisting of a hydrogen atom only is a ceramide. Glc: glucose; Gal: galactose; NeuNAc: N-acetylneuraminic acid (i.e. sialic acid). GA stands for asialo-, GM for monosialo-, GD for disialo-, GT for trisialo-, GQ- for quadrisialo- and GP for pentasialo-ganglioside.

Glycosphingolipids were discovered by the German scientist Ernst Klenk after their isolation from brain tissue in 1942 [1]. They are amphipathic molecules composed of a sphingosine base to which are linked a fatty acid chain and a hydrophilic monosaccharide or oligosaccharide (Fig. 1). Once thought to be relatively inert, glycosphingolipids actually play key cellular roles both as structural components of membranes and as signaling molecules. They have been shown to regulate key cell properties and biological functions such as cell adhesion, cell growth, cell proliferation, autophagy, apoptosis and senescence [2]. Increasing evidence suggests that glycosphingolipids are also potent regulators of the inflammatory process. For instance, defects in the catabolism of glycosphingolipids in human inherited lysosomal storage disorders or in experimental murine models frequently cause neuroinflammation, along with progressive neurodegeneration. While lysosomal storage disorders are rare diseases, changes in glycosphingolipid homeostasis are also evidenced in common neurodegenerative diseases such as Parkinson’s disease. In the present review, we consider the links between glycosphingolipids and neuroinflammation in Parkinson’s disease. After outlining the immune-mediated mechanisms involved in Parkinson’s disease, we present the metabolism of glycosphingolipids and their role in the central nervous system. We next summarize glycosphingolipid-related genetic associations and central and peripheral changes in Parkinson’s disease and discuss the role of glycosphingolipids in neuroinflammation based on insights from human inherited lysosomal storage disorders and corresponding experimental models. Finally, we highlight key mechanisms by which glycosphingolipid metabolism alterations could contribute to neuroinflammation in Parkinson’s disease such as inflammasome activation and secretion of pro-inflammatory cytokines, altered calcium homeostasis, changes in the blood-brain barrier permeability, recruitment of peripheral immune cells or production of autoantibodies.

### Neuroinflammation in Parkinson’s disease

**Fig. 2:**
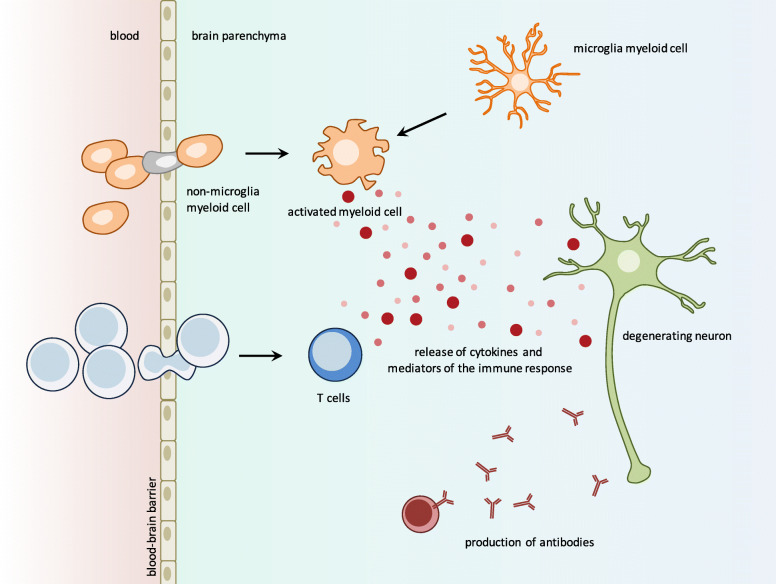
Neuroinflammation and considered immune-mediated mechanisms in Parkinson’s disease. Both innate (e.g. microglia and blood-born myeloid cells activation) and adaptive (e.g. T cells recruitment and antibodies production) immune responses could contribute to neuroinflammation

Parkinson’s disease is the most prevalent movement disorder in elderly adults. It is characterized by the progressive degeneration of dopaminergic neurons in the *substantia nigra* and by the pathological accumulation of alpha-synuclein-immunopositive intracellular aggregates that consist of crowded organelles and lipid membranes [3, 4]. The molecular mechanisms leading to neurodegeneration likely implicate numerous processes both inside degenerating neurons (cell autonomous) and outside degenerating neurons (non-cell autonomous) in other neuronal and non-neuronal cell types. The identification of genetic determinants associated to Parkinson’s disease has led to the proposition that abnormal processing of aberrant or misfolded proteins, mitochondrial dysfunction, disruption of the autophagy-lysosome system, endoplasmic reticulum stress, dysregulation of calcium homeostasis may contribute to the deterioration of dopaminergic neurons [5, 6]. Several evidences also suggest important roles of neuroinflammation and immune-mediated mechanisms. First, classical activation of microglial cells, the resident mononuclear phagocytes of the central nervous system [7], is constantly observed in the brain of patients with Parkinson’s disease [8]. Second, specific *HLA* variants *HLA-DRA* and *HLA-DRB1* are associated to the etiology of Parkinson’s disease [9, 10]. Third, microglial cell activation can be triggered experimentally by Parkinson’s disease-causing gene products such as alpha-synuclein [11, 12]. Besides microglia, non-microglia myeloid cells including macrophages (mature forms of monocytes) and lymphoid cells could also play a role. T cells have been detected in the central nervous system of patients with Parkinson’s disease [13, 14]. This infiltration of lymphocytes into the brain is not likely to be a generalized nonspecific leucocyte response as brain staining for cluster of differentiation (CD) 8 (cytotoxic T cells) and CD4 (helper T cells) was detected while staining for CD57 (natural killer cells) and CD79 alpha and CD20 (B cells) was absent in postmortem human Parkinson’s disease brains [13]. Although they have not been identified as infiltrating lymphocytes [13], a potential role for B cells is also considered [15] and for example deposits of immunoglobulin G are found on dopaminergic neurons and immunolabel Lewy bodies in patients with Parkinson’s disease [16]. The neuroinflammation and immune-mediated mechanisms considered in Parkinson’s disease are outlined in Fig. 2. Although microglia activation is required to guarantee central nervous system integrity, it has become clear that unregulated inflammatory responses and the release of pro-inflammatory cytokines and reactive oxygen species are responsible for neurotoxic processes, for example decreasing neurogenesis [17], causing neuronal loss [18] and altering neuronal patterns of synaptic plasticity [19]. To date, a major challenge is to identify targets for manipulating the immune responses in neurodegenerative disorders. Various pro-inflammatory cytokines including interleukin (IL)-1beta, IL-6, and tumor necrosis factor (TNF)-alpha and anti-inflammatory cytokines such as IL-10, TGF-beta, IL-11 are rightfully considered as potential biomarkers or therapeutic targets [20]. However other molecular players could modulate neuroinflammation. This is the case of glycosphingolipids that metabolism alterations are associated to neuroinflammation both in inherited lysosomal storage disorders and in Parkinson’s disease.

### Metabolism of glycosphingolipids

The simplest glycosphingolipids are glucosylceramides (GlcCer) and galactosylceramides (GalCer), together referred to as monoglycosylceramides or cerebrosides. Their de novo synthesis requires the generation of ceramides and, respectively, the subsequent addition of glucose or galactose residues by the UDP-glucose ceramide glucosyltransferase (UGCG; responsible for the synthesis of glucosylceramides) or the UDP glycosyltransferase 8 (UGT8; responsible for the synthesis of galactosylceramides). Galactosylceramides can be further processed by the addition of galactose (generating galabiosylceramide; GalGalCer), sialic acid (generating GM4; NeuAcGalCer), or sulfate (generating SM4; SGalCer). Glucosylceramides can be converted into lactosylceramides (LacCer) by addition of galactose catalyzed by the B4GALT6 enzyme. The glycosylation of lactosylceramide is then a decisional point towards the production of other glycosphingolipids. Lactosylceramides serve indeed to the productions of GA2 (through the B4GALNT), GM3 (through the addition of sialic acids by ST3GAL5), GB3 (globotriaosylceramide; through the A4GALT) and LC3 glycosphingolipids (through the actions of B3GNT5). GA2, GM3, GB3 and LC3 are then precursors for the synthesis of more complex glycosphingolipids belonging to the asialo-series, ganglio-series, globo/iso-globo-series and lacto/neo-lacto-series, respectively. We refer the reader to the review of Sandhoff and Sandhoff for more detailed information on ganglioside metabolism [21]. Matured glycosphingolipids are transferred via vesicular transport to the plasma membranes outer layer where they cluster in specific lipid membrane microdomains (lipid rafts, domains enriched in cholesterol and sphingolipids). This organization has been proposed to modulate the functional features of several membrane proteins through the maintenance of a dynamic membrane organization or through direct specific lipid-to-protein interaction [22].

**Fig. 3:**
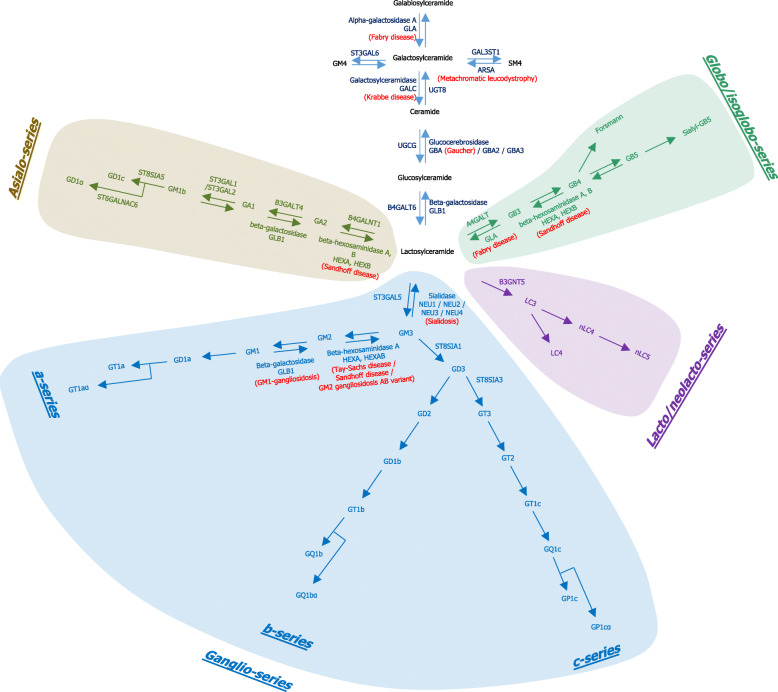
Biosynthetic and degradation pathways of glycosphingolipids in the brain. The nomenclature for gangliosides and the components are based on those of Svennerholm [23] and IUPAC-IUBMB Joint Commission on Biochemical Nomenclature [24]. Official Gene symbol and Full Name are from HUGO (http://www.genenames.org/. Accessed June 28 2019). Glycosphingolipids belonging to the asialo-series, ganglio-series, lacto/neo-lacto-series and globo/iso-globo-series are colored in brown, blue, purple and green, respectively. Lysosomal glycosphingolipid storage disorders resulting from an enzyme defect are indicated in brackets and in red in the figure. The abbreviations are as follows: A4GALT alpha 1,4-galactosyltransferase (22q13.2); ARSA arylsulfatase A (22q13.33); B3GALT4 beta-1,3-galactosyltransferase 4 (6p21.32); B4GALNT1 beta-1,4-N-acetyl-galactosaminyltransferase 1 (12q13.3); B4GALT6 beta-1,4-galactosyltransferase 6 (18q12.1); GAL3ST1 galactose-3-O-sulfotransferase 1 (22q12.2); GALC galactosylceramidase (14q31.3); GBA glucosylceramidase beta also named GBA1 (1q22); GBA2 glucosylceramidase beta 2 (9p13.3); GBA3 glucosylceramidase beta 3 (4p15.2); GLA galactosidase alpha Xq22.1; GLB1 galactosidase beta 1 (3p22.3); HEXA hexosaminidase subunit alpha (15q23); HEXB hexosaminidase subunit beta (5q13.3); NEU1 neuraminidase 1 (6p21.33); NEU2 neuraminidase 2 (2q37.1); NEU3 neuraminidase 3 (11q13.5); NEU4 neuraminidase 4( 2q37.3); ST3GAL1 ST3 beta-galactoside alpha-2,3-sialyltransferase 1 (8q24.22); ST3GAL2 ST3 beta-galactoside alpha-2,3-sialyltransferase 2 (16q22.1); ST3GAL5 ST3 beta-galactoside alpha-2,3-sialyltransferase 5 (2p11.2); ST3GAL6 ST3 beta-galactoside alpha-2,3-sialyltransferase 6 (3q12.1); ST6GALNAC6 ST6 N-acetylgalactosaminide alpha-2,6-sialyltransferase 6 (9q34.11); ST8SIA1 ST8 alpha-N-acetyl-neuraminide alpha-2,8-sialyltransferase 1 (12p12.1); ST8SIA3 ST8 alpha-N-acetyl-neuraminide alpha-2,8-sialyltransferase 3 (ST8SIA3, 18q21.31); ST8SIA5 ST8 alpha-N-acetyl-neuraminide alpha-2,8-sialyltransferase 5 (18q21.1); UGCG UDP-glucose ceramide glucosyltransferase (9q31.3); UGT8 UDP glycosyltransferase 8 (4q26)

Removal of sphingolipids from the plasma membrane occurs through the endolysosomal pathway. Upon incorporation into the lysosomes, glycosphingolipids are degraded into monoglycosylceramides and then ceramides by the action of several glycosidases. Deficiency in one enzyme or co-factor results in the complete blockage of the catabolic chain and in the accumulation of undegraded material in lysosomes, as observed in lysosomal glycosphingolipid storage disorders. For instance, complex glycosphingolipids are hydrolyzed to glucosylceramides and galactosylceramides through the activity of enzymes such as alpha-galactosidase A (encoded by the *GLA* gene; that deficiency causes Fabry disease), beta-galactosidase (encoded by *GLB1*; that deficiency causes GM1 gangliosidosis), sialidases (*NEU1*, *NEU2*, *NEU3* or *NEU4* ; associated to the sialidosis) or beta-hexosaminidase A and B (that deficiencies are associated with mutation in the *HEXB* gene responsible for Sandhoff disease or with mutation in the *HEXA* gene responsible for Tay-Sachs disease). Glucosylceramides and galactosylceramides are then hydrolyzed respectively by glucocerebrosidase (also named glucosylceramidase; the *GBA* gene encoding lysosomal glucocerebrosidase is associated to Gaucher disease) and galactosylceramidase (encoded by the *GALC* gene; that deficiency causes Krabbe disease) to regenerate ceramides. Ceramides are further deacetylated to sphingosines that can be broken down or recycled for sphingolipid synthesis by the salvage pathway [25]. Non-lysosomal pathways of degradation also exist and for instance glucosylceramides can be degraded not only by the lysosomal glucocerebrosidase, but also by the non-lysosomal glucocerebrosidase (encoded by the *GBA2* gene) and the cytosolic Klotho-related glucocerebrosidase (encoded by the *GBA3* gene) [26–31]. Fig. 3 depicts the biosynthetic and degradation pathways of glycosphingolipids in the brain and can be used by the reader to follow the biochemical pathways discussed throughout the review.

### Glycosphingolipids in the central nervous system

The nervous system is among the tissues in the mammalian body that have the highest lipid content as well as the highest lipid complexity [32]. Glycosphingolipids in the brain are especially abundant, complex and derive mostly from glucosylceramides, although some are derived from galactosylceramides. In the adult mammalian brain, the four major brain sphingolipids are the gangliosides of the a- and b-series GM1, GD1a, GD1b and GT1b that have a hydrophilic tetraosyl moiety with one to four sialic acids [21, 33–36]. The grey matter and neurons are particularly enriched in gangliosides, while oligodendrocytes and myelin are highly enriched in galactosylceramides and their sulfated derivates sulfatides [37]. This complexity is increased manifold when is taken into consideration that (i) the sphingolipid profile of the brain continuously changes as the brain develops and ages [35, 37–39], (ii) the changes in sphingolipid composition can be highly regional and (iii) cell-specific glycosphingolipids patterns and metabolism have been observed for different neurons, including granule neuron, pyramidal neurons, and Purkinje cells [21]. The tight regulation of glycosphingolipid metabolism during embryonic development suggests a critical role for glycosphingolipids during brain development and we refer the reader to the review of Furukawa and colleagues for this aspect [40]. Glycosphingolipids have also been shown to have structural functions in the adult brain both in maintaining the integrity of cellular and sub-cellular compartments and in regulating intra- and inter-cellular signaling activities involved in cell polarity [41], neuronal differentiation, synapse formation, synaptic transmission, energy metabolism [42], insulin resistance [43] and glial–neural interactions [44–46]. At the molecular level, glycosphingolipids have been shown to modulate the activity of membrane-associated proteins including receptor-associated tyrosine kinases [47–50] and non-receptor tyrosine kinases of the Src family [51–53]. For example GM3 gangliosides inhibit epidermal growth factor receptor signaling and insulin receptor signaling and these inhibitions are mediated, at least in part, by the specific interaction of GM3 with these receptors [54, 55]. Of note, the four major brain gangliosides GM1, GD1a, GD1b and GT1b all increase significantly from 5 months of gestation to 5 years of age. After 5 years of age, the proportion of GM1 and GD1a decreases, while the proportion of GM3, GD3, GT1b and GD1b increases [56, 57]. To what extend these changes contribute to the susceptibility of the brain to age-related neurodegenerative diseases remains unknown.

### Glycosphingolipid metabolism in Parkinson’s disease

#### Glycosphingolipid-related genetic determinant of Parkinson’s disease

The relationship between glycosphingolipids and Parkinson’s disease has with no doubt gained attention since carrying a mutated allele of the *GBA* gene encoding the lysosomal glucocerebrosidase was identified as one of the most frequent genetic risk factor for Parkinson’s disease (carrying two mutated copies of *GBA* causes Gaucher disease) [58–60]. So far 300 pathogenic mutations have been identified throughout the *GBA* gene, with N370S (c.126A > G; p.Asp409Ser current nomenclature) being the most common mutation followed by the L444P (c.1448T > C; p.Leu483Pro current nomenclature) [61]. A link between glycosphingolipids in Parkinson’ s disease is also supported by the association to Parkinson’s disease of other genes related to glycosphingolipid storage disorders. Among these are the scavenger receptor class B member 2 (*SCARB2*) gene that encodes the lysosomal integral membrane protein type-2 (LIMP2) that is involved in the delivery of glucocerebrosidase to lysosomes [62–66], as well as the CTSD gene encoding Cathepsin D that is a lysosomal protease involved in the posttranslational cleavage of prosaposin thus leading to the production of the glucocerebrosidase activator saposin C [67]. Furthermore, it should be noted that *CTSB* (cathepsin B), *GALC* (galactosylceramidase) and *VPS35* (that encodes a retromer subunit) that are associated to Parkinson’s disease and *PLA2G6* that is the causative gene for early-onset PARK14-linked dystonia-parkinsonism could also influence sphingolipid metabolism as observed in experimental models [68–71].

#### Central deregulation of glycosphingolipid metabolism in Parkinson’s disease

Glycosphingolipid metabolism has been analyzed in brain samples from patients with Parkinson’s disease. Several studies reported decreased glucocerebrosidase activity in the brain of patients with and without *GBA* mutations, as compared to control individuals with the more pronounced decrease often reported in the *substantia nigra* [72–76]. Interestingly, a gradual reduction in glucocerebrosidase activity was also evidenced with aging, suggesting that a decreased brain glucocerebrosidase activity may contribute to the age-related risk for neurodegeneration [75, 77]. A more recent study also reported a decreased activity of alpha-galactosidase A in Parkinson’s disease (encoded by the *GLA* gene that deficiency is associated to Fabry disease) [78]. Also, a decrease in the gene expression of enzymes involved in the synthesis of GM1 and GD1b (*B3GALT4*) and GD1a and GT1b glycosphingolipids (*ST3GAL2*) were also reported in residual neuromelanin-containing cells in the *substantia nigra* of Parkinson’s disease patients compared to age-matched controls [79].

With respect to glycosphingolipids levels, a lipidomic analysis carried out by Gegg and collaborators reported no changes in the amounts of glucosylceramides or lactosylceramides or gangliosides in either of putamen or cerebellum from patients with Parkinson disease with or without *GBA* mutation, although a trend was seen for increased GM2 and GM3 gangliosides in the putamen of patients carrying a mutated allele of *GBA* [80]. In another study, Hadaczek and collaborators used high-performance thin-layer chromatography and reported decreases in GM1, GD1a, and GD1b levels in the occipital cortex of Parkinson’s disease patients as well as decreases in GD1a, GD1b, and GT1b levels along with a smaller magnitude decrease in GM1 that did not reach statistical significance in the *substantia nigra* from Parkinson’s disease patients [81]. A decrease in GM1 in Parkinson’s disease patients is also supported by the study of Wu and coworkers who evidenced using FITC-cholera toxin B histochemistry that only approximately 20% of *substantia nigra* dopamine neurons the expressed GM1 in patients compared to almost 62% in control individuals.

Although the aforementioned data are difficult to compare given their differences in the glycosphingolipids and samples examined and the severity of the disease, they support that alterations in the biosynthesis or catabolism of glucosylceramides or downstream glycosphingolipids occur in the brain of patients with Parkinson’s disease.

#### Deregulation of glycosphingolipid metabolism in the cerebrospinal fluid and blood of patients

Glycosphingolipid metabolism has also been studied in cerebrospinal fluid and blood samples from patients. Decreased protein levels and enzymatic activity of glucocerebrosidase were reported in the cerebrospinal fluid of patients with Parkinson’s disease independently of their *GBA* mutation carrier status [82–84], although such decrease was not significant in all studies [85]. Decreased glucocerebrosidase activity could also be detected in dried blood spots [86, 87], peripheral blood mononuclear cells [88] and monocytes but not in lymphocytes [89] of patients. Alteration in the activity of other enzymes involved on glycosphingolipid metabolism were also reported in cerebrospinal fluid of patients with Parkinson’s disease including higher cathepsin E and beta-galactosidase activity [85] and decreased beta-hexosaminidase activity [82, 84].

Regarding changes in glycosphingolipids levels, Mielke and collaborators reported in a pilot study that levels of monohexylceramides (e.g. glucosylceramides and galactosylceramides) and lactosylceramides were higher in the plasma from Parkinson’s disease patients compared to controls [90]. More recently, Chan and coworkers analyzed the lipidomic profile of plasma obtained from 150 idiopathic Parkinson’s disease patients and 100 controls, measuring 520 lipid species from 39 lipid subclasses including all major classes of glycerophospholipids, sphingolipids, glycerolipids and sterols and reported elevated GM3 gangliosides plasma concentration as the most significant difference between Parkinson’s disease and controls [91].

**Fig. 4:**
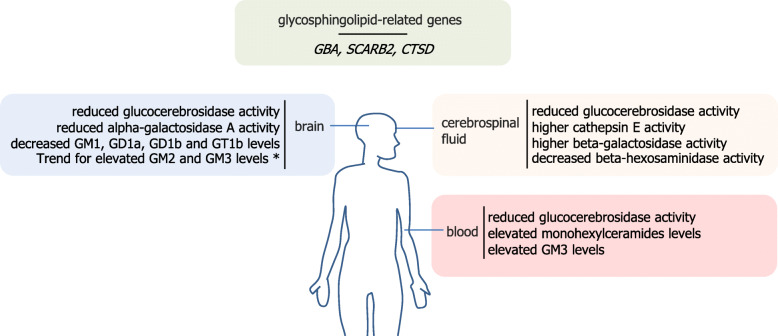
Glycosphingolipid-related genes and deregulations of glycosphingolipid metabolism in Parkinson’s disease. Observations marked with an asterisk (*) have only been reported in patients carrying *GBA* mutation.

Taken together, glycosphingolipid-related genetic determinants of Parkinson’s disease and central and peripheral metabolic changes in patients strongly suggest that glycosphingolipids contribute to Parkinson’s disease pathogenesis (Fig. 4). Thus, Parkinson’s disease may share some disease-related molecular pathways with lysosomal glycosphingolipid storage disorders that also frequently present with neuroinflammation and neurodegeneration.

### Glycosphingolipids and neuroinflammation: insights from lysosomal glycosphingolipid storage disorders

Lysosomal storage disorders are a class of metabolic disorders caused by genetic defects in proteins that are critical for lysosomal function. Approximately 70 different monogenic autosomal or X-linked lysosomal storage disorders are known and are usually classified according to the nature of the principal accumulating substrate. Over two-thirds of lysosomal storage disorders (including those caused by an accumulation of substrates other than glycosphingolipids) involve central nervous system dysfunction leading for example to progressive cognitive and motor decline and these symptoms are often the most debilitating [92]. Among lysosomal storage disorders, those resulting from an abnormal build-up of undegraded glycosphingolipids and their respective defective enzyme are Gaucher disease (ORPHA355; the most common lysosomal storage disorders) caused by deficiency of glucocerebrosidase, related to the presence of two disease-causing alleles in the *GBA* gene with accumulation of glucosylceramides; GM1 gangliosidosis (OMIM 230500) caused by deficiency of beta-galactosidase-1, related to mutations in the *GLB1* gene; GM2 gangliosidosis caused by deficiency of the beta-hexosaminidase enzyme itself or GM2-activator protein (all necessary for degradation of GM2-ganglioside) either due to autosomal recessive gene defects in *HEXA* for Tay-Sachs disease (OMIM 272800), *HEXB* for Sandhoff disease (OMIM 268800) or *GM2A* for GM2-gangliosidosis AB variant (OMIM 272750); Fabry disease (OMIM 301500; the second most common lysosomal storage disorders) caused by deficiency of alpha-galactosidase A, related to mutations of the *GLA* gene, with accumulation of GB3 glycosphingolipids. Most of glycosphingolipid storage disorders display a broad spectrum of clinical severity that likely reflects the loss of enzymatic activity, ranging from infantile (little or no enzymatic function) to juvenile and adult forms (moderate or mild residual enzymatic function). Pathology in the central nervous system appears as a common feature of glycosphingolipid storage disorders. For example, Gaucher disease results in the progressive accumulation of glucosylceramides in lysosomes of cells of the monocyte/macrophage lineage, inducing their transformation into Gaucher cells, enlarged cells with aggregates of undegraded glucosylceramides. Three clinical forms are classically distinguished: Gaucher disease type 1 (OMIM 230800), which affects the majority of patients, shows a variety of systemic manifestations, including hepatosplenomegaly, anemia, thrombocytopenia, and skeletal complications related to bone marrow infiltration by Gaucher cells, whereas Gaucher disease types 2 (OMIM 230900) and 3 (OMIM 231000) are classified as neuronopathic Gaucher as they are associated respectively with severe or variable neurologic manifestations in addition to above features [93, 94]. While Gaucher disease type 1 has been historically distinguished by the lack of neurologic manifestations, it is now known that it is in fact associated with a 4-9% risk of developing Parkinson’s disease, a risk comparable to that of people carrying a single mutant allele of *GBA* [95]. Even when Parkinson’s disease criteria are not met, over 21% of patients with Gaucher disease type 1 may exhibit at least one parkinsonian finding [96], suggesting that the three forms of Gaucher disease each involve a different profile of neurological manifestations [94, 97]. It should be noted that parkinsonism has also been reported in patients affected by adult-onset GM2 gangliosidosis [98–100] and in patients affected with Fabry diseases [101], although the association between Parkinson’s disease and Fabry disease should be considered with caution [102, 103]. A neuropathological relationship between glycosphingolipid storage disorders and Parkinson’s disease is also supported by reports of alpha-synuclein-immunopositive intracellular aggregates in the brain patients with Gaucher disease [104, 105] and GM2 gangliosidosis [106]. Gaucher disease also shows striking alterations in the immune system. Microgliosis has been noted along with neuronal loss and astrogliosis in Gaucher disease types 2 and 3 [104, 107, 108]. Increased serum levels of cytokines such as IL-1beta, IL-1 receptor antagonist, IL-6, TNF-alpha, and soluble IL-2 receptor levels were reported in Gaucher disease type 1 patients compared to normal controls [109]. Also, Zahran and colleagues used three-color flow cytometric immunophenotyping for determination of the frequency of lymphocyte subpopulations and activated T lymphocytes in the peripheral blood among eighteen children with Gaucher disease type 1. They showed increases in the frequencies of activated T lymphocytes (CD3+HLA-DR+), activated T-helper cells (CD4+HLA-DR+) and activated T-suppressor/cytotoxic cells (CD8+HLA-DR+) in Gaucher disease type 1 as compared to healthy children [110]. Shoenfeld and coworkers demonstrated a significant increase in the incidence of autoantibodies against 14 autoantigens in the sera of 43 patients with Gaucher disease, ranging from 11% for anti-ribonucleoprotein, anti-pyruvate dehydrogenase and anti-DNA antibodies to 57% for rheumatoid factor [111]. In addition, elevated levels of pro-inflammatory cytokines such as TNF-alpha were also reported in the cerebrospinal fluid of patients with Tay-Sachs [112, 113]. Fabry disease, the second most common lysosomal storage disorders have also been associated with an higher pro-inflammatory cytokine expression and production and a probable role of the toll-like receptor (TLR) 4 and CD1d pathways triggered by GB3 accumulation has been proposed [114–116]. Also, Martinez and coworker evaluated in a series of patients with Fabry disease the prevalence of autoantibodies against extractable nuclear antigens, double-stranded DNA, anticardiolipin and phosphatidylserine disease and reported that 57% of the samples showed reactivity with at least one autoantigen [117]. Altogether, these studies support that a wide range of glycosphingolipids storage disorders present with neuroinflammation and immune dysregulation such as increase of pro-inflammatory cytokines and chemokines, changes in the proportion of activated T lymphocytes and/or production of autoantibodies [118]. This relationship between glycosphingolipids and neuroinflammation has been further explored through the characterization of gene-targeted mouse models.

### Glycosphingolipids and neuroinflammation: insights from gene-engineered animals

Several mouse strains have been generated based on the disruption of enzymes involved in the catabolism of glucosylceramide or downstream glycosphingolipids and their characterization helps to gain insight into the role of glycosphingolipids in neurodegeneration and neuroinflammation. These include models deficient for *GBA* (e.g. modeling Gaucher disease), *HEXB* (e.g. Sandhoff disease) or *HEXA* (e.g. Tay-Sachs) or *GLB1* (for GM1 gangliosidosis). Among the mouse models disrupted for glucocerebrosidase, the Gba^flox/flox^; nestin-Cre mouse model in which glucocerebrosidase deficiency is restricted to neurons and macroglia, with normal glucocerebrosidase activity in microglia, exhibit rapid motor dysfunction including rigidity of limbs and abnormal gait, leading to seizures and paralysis by 21 days of age, at which time mice exhibit massive microglial activation, astrocytosis and neuronal loss [119]. The time course analysis of the neuropathological changes in this model show that microglia activation precedes neuronal loss in defined areas as well as the onset of noticeable symptoms [120]. Consistently, elevated transcript levels of the pro-inflammatory mediators including IL1-1beta, TNF-alpha, TNFR-1, Colony Stimulating Factor 1 and transforming growth factor-beta, chemokine (C-C motif) ligand (CCL)2, CCL3 and CCL5 and reactive oxygen species are detected early in the brain of this mouse model and correlate with the progression of the pathology [121, 122]. Likewise, microglial activation and elevation of pro-inflammatory factors have been reported in the Hexb^−/−^ mouse model of Sandhoff disease and this pro-inflammatory phenotype also likely precedes the neurodegeneration in this model [100, 123–125]. Studies also demonstrated that macrophage inflammatory protein (MIP)-1alpha is upregulated both in the brains and in microglial cells derived from Hexb^−/−^ model mice [125–127]. Microglia activation has also been consistently been reported in GM1 gangliosidosis mice [124], further supporting that alterations in various glycosphingolipids metabolic pathways cause central nervous neuroinflammation. As aforementioned, this neuroinflammation can precede the neurodegeneration and the onset of symptom suggesting a potentially contributory role in the disease progression. Supporting this hypothesis, the genetic deletion of the immune mediators MIP-1 or TNF-alpha reduced neurodegeneration and increased lifespan in Hexb^−/−^ mice [128]. Moreover, the improved neurological outcomes observed after fetal gene therapy in glucocerebrosidase deficient mice [129] or substrate reduction therapy or bone marrow transplantation in beta-hexosaminidase -/- mice [100, 124] were achieved with a down-regulation of several inflammatory markers such as microglia numbers. Taken together, human and experimental data support that glycosphingolipid contribute to immune-mediated mechanisms contributing to the establishment of chronic neuroinflammation.

### Putative mechanisms linking glycosphingolipids and neuroinflammation in Parkinson’s disease

As presented in the first paragraph, several immune-related mechanisms are considered to participate to the neuroinflammatory process in Parkinson’s disease. These include classical activation of myeloid microglial cells typified by the production of inflammatory cytokines, infiltration of peripheral immune cells within the brain parenchyma and production of immunoglobulins. The finding that glycosphingolipid metabolism is altered in Parkinson’s disease and that such alterations are consistently associated to neuroinflammation in lysosomal storage disorders and experimental models led us to further consider putative mechanisms linking glycosphingolipids and neuroinflammation.

#### Glycosphingolipids, activation of the inflammasome and secretion of pro-inflammatory cytokines

**Fig. 5:**
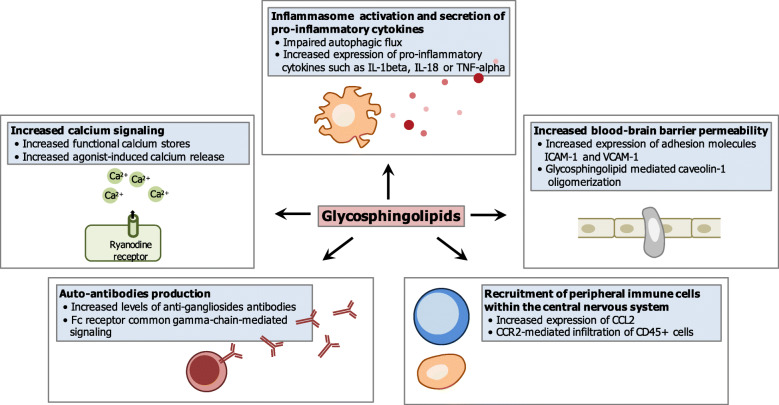
Putative mechanisms linking glycosphingolipids and neuroinflammation in Parkinson’s disease. Glycosphingolipid participation to neuroinflammation in neurodegenerative diseases could involve multiple mechanisms: autophagy impairments and inflammasome activation with the secretion of pro-inflammatory cytokine IL-1beta and IL-18; increased functional Ca^2+^ stores and increased agonist-induced Ca^2+^ release; increased blood–brain barrier permeability with increased expression of adhesion molecules ICAM-1 and VCAM-1 and caveolin-1 oligomerization; increased production of CCL2 and infiltration of CCR2+ peripheral immune cells; evocation of autoimmune responses and production of anti-glycosphingolipid autoantibodies.

Inflammasomes are intracellular multimeric protein complexes that upon assembly cleave the pro-inflammatory caspase-1 into its enzymatically active form that is responsible for the maturation of the pro-inflammatory cytokines IL-1beta and IL-18 [130, 131]. Inflammasomes assemble in response to wide-ranging stimuli, including pathogen-associated molecular patterns (PAMPs) and damage-associated molecular patterns (DAMPs) [11, 132]. Hence, high levels of IL-1beta and IL-18 can be detected in Parkinson’s disease and are considered to be crucial for the establishment of a chronic neuroinflammation (for review, see [133]). Autophagy, a dynamic cellular process involved in the degradation of damaged organelles, denatured proteins or invading pathogens through a lysosomal degradation pathway, also intervene in the regulation of inflammasome activation. For instance, autophagic removal of intracellular DAMPs, inflammasome components or cytokines can reduce inflammasome activation [130]. Aflaki and coworkers characterized macrophages derived from peripheral monocytes from patients with type 1 Gaucher disease and reported persistent activation of inflammasomes leading to the maturation of IL-1beta in these cells [134]. Treatment with the small-molecule glucocerebrosidase chaperone NCGC758 reversed these defects, inducing autophagy and reducing IL-1beta secretion. Consistently, the inhibition of glucocerebrosidase enzymatic activity by the pharmacological compound conduritol-beta-epoxide in THP-1 cells (human monocytic cell line) led (i) to impaired autophagic flux associated with a defective efferocytosis and (ii) to inflammasome activation with increased maturation of IL-1beta [135]. A role for lactosylceramides has also been discovered in the regulation of cytokine-induced expression of proinflammatory mediators in rat primary astrocyte-enriched cultures. In this model, silencing of the lactosylceramide synthase gene through the use of antisense oligonucleotides decreased lipopolysaccharide/ interferon (IFN)-gamma-induced inducible nitric oxide synthase (iNOS), TNF-alpha, and IL-1beta gene expression and this was reversed by lactosylceramides supplementation [136]. Thus, these reports document a tight link between glycosylceramides and lactosylceramides with the regulation of expression of mature pro-inflammatory cytokines that are crucial in the establishment of chronic neuroinflammation (Fig. 5).

#### Glycosphingolipids, altered calcium homeostasis and neuroinflammation

Calcium is a highly versatile intracellular signal that is essential to intracellular signaling and intercellular communication to regulate many different cellular processes [137]. Elevated intracellular levels of calcium ions (Ca^2+^) were reported in series of neurodegenerative diseases including Parkinson’s disease and glycosphingolipid storage disorders such as Gaucher disease [138–140]. Studies showed that glucosylceramides increase functional calcium stores in cultured neurons [141] and agonist-induced calcium release from intracellular stores via the ryanodine receptor [138]. In line, agonist-induced calcium release via the ryanodine receptor was significantly enhanced in brain microsomes from the acute neuronopathic form of Gaucher disease (type 2) and correlated with glucosylceramides accumulation [142]. Altered Ca^2+^ signaling and homeostasis can induce multiple inflammatory processes in many types of cells in the brain. In the case of microglia, elevated Ca^2+^ can induce inflammasome activity, cytokine release, and NF-kappaB activation. Some inflammatory-related mediators, such as the S100 family members, are themselves Ca^2+^-binding proteins that exhibit increased activity in the presence of Ca^2+^ [143]. Other inflammatory mediators are indirectly induced by Ca^2+^ through the activation of a variety of signaling cascades involving calcium/calmodulin-dependent protein (CaM) kinase and protein kinase C (PKC) among others. On this basis, Ca^2+^ signaling is another mechanism by which glucosylceramides accumulation could contribute to neuroinflammation in Parkinson’s disease.

#### Glycosphingolipids and changes in the blood-brain barrier permeability

The blood-brain barrier is a physical barrier formed by endothelial cells via interactions with pericytes and astrocytes that prevents blood proteins, antibodies and immune cells from penetrating into the brain parenchyma [144]. Post-mortem tissue analyses and neuroimaging studies show that the blood-brain barrier can be compromised in Parkinson’s disease [145, 146]. Several lines of evidence support glycosphingolipids could regulate this blood-brain barrier permeability. First, blood–brain barrier permeability is increased in mouse models disrupted for glucocerebrosidase (Gba^flox/flox^; nestin-Cre; modeling Gaucher disease), beta-hexosaminidase mice (modeling Sandhoff disease) or beta-galactosidase-1 (modeling GM1 gangliosidosis), as evidenced with gadolinium diethylenetriaminepentaacetic acid or Evans blue extravasation [121, 124]. Second, silencing of the lactosylceramide synthase gene in TNF-alpha and IFN-gamma-stimulated astrocytes attenuates the expression of adhesion molecules (e.g., intercellular adhesion molecule (ICAM)-1 and vascular cell adhesion molecule (VCAM)-1) that bind and recruit circulating T cells and monocytes. This is reversed by the addition of lactosylceramide [147]. Third, the depletion of glycosphingolipids by treatment with a series of sphingolipid synthesis inhibitors in cultured ECV-304 cells and Hela cells disrupt caveolin-1 high molecular weight oligomer formation [148] that promotes the transport of immune cells and antibodies across the barrier [149]. Taken together, these data suggest that alterations in glycosphingolipid metabolism may increase the blood-brain barrier permeability and render the brain susceptible to the infiltration of peripheral myeloid and lymphoid cells, antibodies and pro-inflammatory cytokines that would otherwise be restricted from the brain.

#### Glycosphingolipids and the recruitment of peripheral immune cells within the central nervous system

As mentioned above, the recruitment into the brain parenchyma of peripheral innate and adaptive immune cells can exacerbate neuroinflammation and neurodegeneration. Besides general changes in the blood-brain barrier permeability, recent experimental studies suggest that the infiltration of leucocytes under central nervous system pathological situations occurs through a C-C chemokine receptor (CCR)2 signaling manner [150, 151]. In support of these observation, increased expression of the CCR2 ligand CCL2, also referred to as monocyte chemoattractant protein (MCP)1, is considered as a potential biomarker for Parkinson’s disease [152, 153] and polymorphisms in the *CCL2* gene could be associated to Parkinson’s disease [154]. Clearly, the infiltration of monocytes occurs in lysosomal storage disorders as lipid-engorged macrophages (Gaucher cells) are observed in the brain of Gaucher disease patients [104, 155, 156]. Also, a marked increase in the expression of CCL2 has been documented in bone marrow mesenchymal stromal cells from an adult patient with Gaucher disease type 1 [157] and in cerebrospinal fluids of patients with the severe infantile phenotype of gangliosidoses (Tay-Sachs disease, Sandhoff disease, and GM1-gangliosidosis) [113] as well as in the brain of the Gba^flox/flox^; nestin-Cre [121] and Hexb^−/−^ mouse models [158]. Furthermore, ablation of the chemokine receptor CCR2 in the Hexb^−/−^ mouse results in significant decrease of CD45+ peripheral blood mononuclear cells into the brain, decrease in TNF-alpha and MHC-II mRNA abundance and retardation in clinical disease development [159]. Importantly, CCR2 is also expressed by T cells and regulate the ratio of effector/regulatory T cells [160]. Thus, one can hypothesize that an accumulation of glucosylceramides or GM2 in Parkinson’s disease could participate to the production of CCL2 and to the recruitment of CCR2+ leucocytes into the brain. Noteworthy, glucosylceramides have also been identified as potent activator of invariant natural killer T (innate T lymphocytes that specifically recognize CD1d-bound α-linked glycosphingolipids) [161, 162] further suggesting that glycosphingolipids can contribute to the activation of peripheral immune cells.

#### Glycosphingolipids and the production of autoantibodies

The production and deposition of antibodies is another immune-mediated pathway associated to the establishment of neuroinflammation in Parkinson’s disease [15, 16]. A first study supporting a role for glycosphingolipids in this immune response revealed that the Hexb^−/−^ mouse model of Sandhoff diseases showed an elevation of antiganglioside autoantibodies and that crossing this strain of mice with some deficient in the Fc gamma receptor prolonged their survival, suggesting an antibody-mediated component in this pathological progression [163]. It has subsequently been hypothesized that gangliosides could evoke autoimmune responses due to their impaired degradation and clearance. As a consequence autoantibodies would bind to antigens on the cell surface of neurons and trigger microglial activation via the Fc receptor common gamma-chain [118]. In this regard, various anti-glycosphingolipid autoantibodies have been detected in neurologic disorders including amyotrophic lateral sclerosis [164, 165], Guillain-Barré syndrome [166] and multifocal motor neuropathy [167]. In conclusion, current data show that glycosphingolipid might be targeted by immunoglobulins. The role of anti-glycosphingolipid antibodies in Parkinson’s disease merits further study.

## Conclusions

Data from patients and experimental models strongly suggest that a deregulation in glycosphingolipid metabolism could contribute to neuroinflammation in Parkinson’s disease through inflammasome activation, altered calcium homeostasis, changes in the blood-brain barrier permeability, recruitment of peripheral immune cells or production of autoantibodies (Fig. 5). To date, most insights on the mechanisms linking glycosphingolipids and neuroinflammation arise from experimental models of lysosomal storage diseases that show important enzymatic defects, including Gba^flox/flox^; nestin-Cre and Hexb^−/−^ mouse models. Also, it will be important to further characterize this relationship in mouse models with relatively weak enzymatic effects such as the GBA-heterozygous L444P/wt mice model that appears more relevant to Parkinson’s disease. In this context, it will be particularly interesting to investigate to what extent neuroinflammation is the result of a direct effect of glycosphingolipid deregulation or rather caused by a pathological feedback loop implicating glycosphingolipids and alpha-synuclein accumulation [168, 169]. Glycosphingolipids encompass a diverse family of lipids with diverse structures and properties and roles. As such, identifying the specific glycosphingolipids deregulated and their relative involvement in immune-mediated responses in neurodegenerative diseases is essential. The levels of simple gangliosides such as GM2 and GM3 could be altered in Parkinson’s disease and can potentiate classical microglial activation and the infiltration of peripheral immune cells into the central nervous system. Lactosylceramide synthase (i) is a decisional point towards the production of these simple gangliosides, (ii) is required for the production of pro-inflammatory cytokines in several experimental models and (iii) may play a crucial role in the expression of adhesion molecules and the recruitment immunocompetent cells across the blood-brain barrier. Thus, the inhibition of its enzymatic activity is a potential strategy for modulating the immune responses that could be easily evaluated in mouse models of neurodegeneration. Enhancing the degradation or inhibiting the synthesis of glucosylceramides are other approaches that are achievable in human as demonstrated in patients with Gaucher disease. Ambroxol and LTI-291 (chaperone molecules aimed to increase the level or activity of glucocerebrosidase) and venglustat (a glucosylceramide synthase inhibitor) are now tested in clinical studies for Parkinson’s disease [170]. Importantly, the evaluation of the disease-modifying properties of these molecules should be based not only on synuclein- but also on neuroinflammation-related endpoints. Knowing to what extent glycosphingolipid metabolism and neuroinflammation are modulated by autophagy inducing neuroprotective molecules relying on mTOR inhibition, channel Ca2+ blockers or targeted Transcription Factor EB overexpression would also be exciting [171, 172]. Finally, the tight link between glycosphingolipids and neuroinflammation reinforce their interest as potential biomarkers. As such, the development of sphingolipidomic or enzyme activity measurements in specific peripheral immune cell populations could help the development of target-engagement assays and diagnostic tools for Parkinson’s disease.

## Data Availability

Not applicable.

## Abbreviations

CCL: Chemokine (C-C motif) ligand
CCR: C-C chemokine receptor
CD: Cluster of differentiation
DAMP: Damage-associated molecular pattern
GLP-1: Glucagon-like peptide-1
ICAM: Intercellular adhesion molecule
IL: Interleukin
INF: Interferon
MCP: Monocyte chemoattractant protein
MIP: Macrophage inflammatory protein (MIP)
PAMP: Pathogen-associated molecular pattern
TLR: Toll-like receptor
TNF: Tumor necrosis factor
UDP: Uridine diphospho
VCAM: Vascular cell adhesion molecule
